# Development of a multi-dimensional measure of resilience in adolescents: the Adolescent Resilience Questionnaire

**DOI:** 10.1186/1471-2288-11-134

**Published:** 2011-10-05

**Authors:** Deirdre Gartland, Lyndal Bond, Craig A Olsson, Simone Buzwell, Susan M Sawyer

**Affiliations:** 1 Healthy Mothers Healthy Families Research Group, Murdoch Children's Research Institute, Melbourne, Australia; 2 Social and Public Health Sciences Unit, Medical Research Council/Chief Scientist Office, Glasgow, UK; 3 Centre for Adolescent Health, Murdoch Childrens Research Institute, Melbourne, Australia: Psychological Sciences and Department of Paediatrics, The University of Melbourne, Melbourne, Australia: School of Psychology, Deakin University, Geelong, Australia; 4 Swinburne Professional Learning, Swinburne University, Melbourne, Australia; 5 Centre for Adolescent Health, Royal Children's Hospital, Melbourne, Australia; Murdoch Children's Research Institute, Melbourne, Australia; Department of Paediatrics, The University of Melbourne, Melbourne, Australia

## Abstract

**Background:**

The concept of resilience has captured the imagination of researchers and policy makers over the past two decades. However, despite the ever growing body of resilience research, there is a paucity of relevant, comprehensive measurement tools. In this article, the development of a theoretically based, comprehensive multi-dimensional measure of resilience in adolescents is described.

**Methods:**

Extensive literature review and focus groups with young people living with chronic illness informed the conceptual development of scales and items. Two sequential rounds of factor and scale analyses were undertaken to revise the conceptually developed scales using data collected from young people living with a chronic illness and a general population sample.

**Results:**

The revised Adolescent Resilience Questionnaire comprises 93 items and 12 scales measuring resilience factors in the domains of self, family, peer, school and community. All scales have acceptable alpha coefficients. Revised scales closely reflect conceptually developed scales.

**Conclusions:**

It is proposed that, with further psychometric testing, this new measure of resilience will provide researchers and clinicians with a comprehensive and developmentally appropriate instrument to measure a young person's capacity to achieve positive outcomes despite life stressors.

## Background

Resilience has been variously defined as positive developmental outcomes in the face of adversity or stress [1,2]; being relatively resistant to psychosocial risk experiences [3], successful adaptation or the development of competence despite high-risk status or chronic stress [4] and the capacity of dynamic systems to withstand or recover from significant disturbances [5]. While differing in terminology, such definitions describe the two common factors necessary for defining resilience; firstly the experience of adversity or stress, and secondly, the achievement of positive outcomes. While resilience research continues to grow, there have been few attempts to integrate current knowledge into measurement tools. In this paper, the development and pilot testing of a comprehensive and theoretically based measure of resilience for adolescents is detailed.

Early research identified resilience as a characteristic of the individual [6], and considered resilient children to be exceptional individuals, unique in their ability to prevail against the odds. Current research now predominantly views resilience as the *process *by which individuals draw on personal characteristics and resources in their environment to enable them to successfully negotiate adversity [1,7-10]. As such, resilience is not seen as a static characteristic of an individual, but rather a dynamic process across contexts and throughout the life span. The process of resilience can be seen as arising from interactions which are central to normal developmental processes that commonly occur and may even be seen as 'ordinary' [1].

A range of factors commonly associated with resilient outcomes have been widely studied and described. While the literature around the concept of resilience is increasing, there have been few attempts to synthesise current research findings into useful measurement tools. The issue with most current measures of resilience is their limited focus, for example, only addressing individual characteristics [11-13]. Other resilience measures have included some environmental factors but are limited in scope and detail [14]. Research findings indicate that resilience is a multi dimensional construct - resilience in one domain does not automatically confer resilience in other domains [15,16]. Thus it is vital to examine resilience more broadly. Other limitations of currently available resilience measures include: a lack of a clearly defined definitions/models of resilience and/or theoretical underpinning; flawed development processes; limited breadth (most cover individual factors only]; and/or inadequate psychometric properties [17]. This paper reports on the development of a new measure of adolescent resilience that: 1) encompasses the full range of individual factors associated with resilient outcomes; 2) includes assessment of resilience factors in the wider social environment; 3) is developmentally appropriate for adolescents; and 4) builds on a clearly defined theoretical framework or model of resilience.

### Development of the Adolescent Resilience Questionnaire

An ecological-transactional model [18,19] was used to provide a conceptual framework for integrating the individual and environmental factors underlying resilience. This model describes an individual's environment as nested levels of increasing proximity - from societal cultural beliefs and values, to neighbourhood and community settings, then family environment and finally the individual [19]. In this model "context and children's functioning are conceptualised as mutually influencing each other" p. 236 [18]. Each level of the environment contains risk and protective factors for the individual and these factors can be transient or enduring. In this context, examination of resilience factors in each nested level is required to develop a comprehensive measure. Salient adolescent ecological 'levels' have been identified as the domains of individual, family, peers, school and community [10,20]. Resilience factors in each of these domains were examined and factors associated with better outcomes for young people facing adversity were identified.

**Figure 1:**
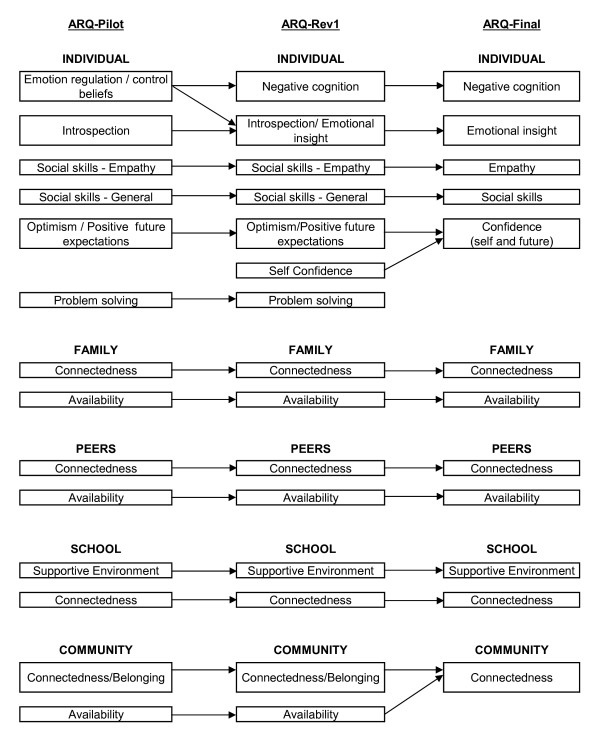
Tracking the ARQ scales through the revision process

A detailed literature review in each of the five domains [17] was supplemented by focus group discussions with adolescents living with a chronic illness recruited from a peer support program [21] and a hospital adolescent ward in order to identify resilience factors to be included in the new measure (see Table 1). Chronically ill adolescents represent an ideal group in which to explore notions of resilience as they face varying levels of adversity in their day to day lives. These adolescents have been shown to be at greater risk of poor outcomes including increased social difficulties, health risk behaviours and mental health states including low self esteem and poor body image [22-27]. However, the majority show positive outcomes, particularly those with less severe illness and without corresponding physical disability [28,29]. Focus group discussions were thematically analysed to derive resilience themes. The primary themes derived from the focus groups fitted well with resilience factors identified in the literature and were developed into 14 conceptual scales across the five domains of self, peers, family, school and community (see Figure 1).

**Table 1:** Study participant numbers, gender and age

	Participants	Female	Age (years)	
	n	%	Range	Mean (SD)
***Focus Groups with chronically ill adolescents***				
Peer support group members				
Metropolitan (3 groups)	14	79	15 - 22	
Regional Victoria (1 group)	6	67	14 - 24	
Hospital ward (1 group)	4	50	19	

Total focus group sample	24	71	14 - 24	18.6 (2.5)

***Pilot testing of the ARQ***				

Catholic secondary school students	330	60	13 - 16	14.3 (0.49)

Adolescents with a chronic illness				
Hospital Clinics				
Asthma	31	58	12 - 17	
Neurology	4	50	15 - 17	
Cystic Fibrosis	73	51	11 - 18	
Rheumatology	23	91	12 - 18	
Adolescent Ward	13	69	13 - 18	
Support Groups				
Diabetes	7	71	14 - 16	
Epilepsy	13	62	14 - 17	
Peer support group (Non illness specific)	40	58	12 - 18	

Total	204	60	11 - 18	14.9 (1.8)

Total pilot testing sample	534	60	11 - 18	14.5 (1.2)

***Revision of the ARQ***				
Random sample secondary schools				
Year seven	191	48	11 - 14	12.4 (0.5)
Year nine	260	51	14 - 17	14.9 (0.6)

Total revision sample	451	50	11 - 17	13.9 (1.4)

The family, peer, school and community domain conceptual scales are self explanatory but some discussion is merited for the individual domain. *Emotion regulation and control beliefs*: Positive emotion regulation skills involve processes by which positive emotionality is maximised or negative emotionality, emotional lability and inappropriate affect minimised. Significant associations have been reported between positive emotion regulation and resilience [30,31], concurring with broader non resilience research supporting the role of emotion regulation and positive outcomes for children [32-34]. A positive association between internal locus of control and resilience has been well documented [35-38]. For example, maltreated children with an internal locus of control were twice as likely to be classified as resilient compared to children with an external locus of control [39].

### Introspection and reflection

This was a strong theme arising from the focus groups that was not identified in resilience literature review. Chronically ill adolescents described the importance of having the time and space to think things through, to work out *what *was happening and *why*, the *meaning *behind events. Adolescents identified this as an important factor in their resilience.

### Social skills (General and Empathy)

Social skills have been described as the interpersonal behaviours needed to develop and deepen supportive personal relationships [40] and have wide ranging implications for healthy development throughout the life span [41,42]. It is unsurprising that social skills have been associated with greater likelihood of resilient outcomes [43]. For example having a positive, reciprocal friendship increased the likelihood of resilience in maltreated children [39]. Resilient young people have been found to have higher empathy and more effective social problem solving skills than those who were stress-affected [36,44].

### Optimism/Positive Future expectations

A positive sense of the future can be conceptualised as "expectations of attaining specific objectives (e.g. achieving in school, having close friends) in later developmental periods" [45] while optimism can be defined as a general expectation of positive outcomes. The two concepts have been shown to be positively correlated and have both been identified as distinguishing resilient children from those affected by stress [7,44,46-50].

### Problem solving

Problem solving abilities have been linked to resilient children compared to their non-resilient peers [35,37,51-53] and have been identified as promoting resilient outcomes in a range of risk situations including poverty and abuse [54], homelessness [31], cancer survivors and parents with a mental illness [55], and depression [56].

A large item pool for each scale was written according to the guiding principals for item development as described by Kline [57] and Streiner and Norman [58]. Consultation with a team of adolescent clinicians and researchers was then used to select the best items for each scale. In this paper, the pilot testing (Study 1) and revision (Study 2) of the newly developed Adolescent Resilience Questionnaire [ARQ] are described. As Study 1 and 2 were identical in method, the common method is reported first and then results for each study are detailed in turn.

## Methods

### Participants

Participants were recruited through Government and Catholic secondary schools, chronic illness support groups, and hospital clinics. Participation required written parent and participant consent.

School students were given a letter explaining the purpose and procedure of the study and parent and participant consent forms. Students who returned signed consent forms completed the paper ARQ questionnaire during class time.

Chronic illness support group members and hospital clinic patients were sent a letter explaining the purpose and procedure of the study, consent forms and the ARQ questionnaire. Completed consent forms and questionnaires were returned separately in the reply paid envelopes provided. After three weeks, reminder letters and replacement forms and questionnaires were sent to all nonrespondents.

Ethics approval was granted from Swinburne University, The Royal Children's Hospital, Department of Education, Employment and Training ethics committees and the Catholic Education Office.

### Materials

The ARQ is a pen and paper questionnaire with scales in five domains: individual, family, peers, school and community. Items comprise statements with a five point Likert response scale labelled: 1 *Never*, 2 *Not often*, 3 *Sometimes*, 4 *Most of the time *and 5 *All the time*. Higher scores indicate greater resilience. A space was available at the end of each domain for participants to write comments regarding items or the questionnaire.

### Analysis

#### Item analysis

Questionnaire items were deleted or revised if they were: identified by respondents as difficult to understand; considered by the researcher to have poor face validity; or endorsed by less than 20% or more than 80% of respondents [26,27].

#### Scale analysis

Factor and reliability analyses were used to guide the construction of scales and selection of the best items. Factor analyses were conducted within each domain with maximum likelihood extraction and oblimin rotation to accommodate possible correlation between factors [28]. Initial eigenvalues and scree plots were employed to select the number of factors. The most parsimonious factor solution was selected according to the following criteria: a good conceptual fit, high percentage of variance explained, high factor loading scores with minimal cross loading and stability of factors across different solutions. Factors were used to construct scales where more than three items loaded at 0.4 or higher and up to eight items were retained per scale. Items loading below 0.3 or on more than one factor were deleted. Internal consistency was assessed using Cronbach alpha. Newly developed scales were examined to ensure fidelity to the original concept, and new items written to fill any gaps in face and content validity or to improve reliability. New items were written following the guiding principals elaborated by Kline [57] and Streiner and Norman [58], drawing on the conceptual underpinning of the scale and taking into account items that had failed to perform during the revision process.

## Results

### Study 1 - Pilot testing of the ARQ

#### Participants

Two samples completed the pilot ARQ. 1) A convenience sample of year 9 students attending Catholic schools in Victoria, Australia. Eleven of the 45 schools invited agreed to participate and 330 of the 1031 eligible students (32% response rate) completed the pilot ARQ (see Table 1). 2) Adolescents with chronic illness were recruited from support groups and hospital clinics. Recruitment from hospital clinics and community support groups ensured the inclusion of adolescents with a range of illness severity. Chronic illnesses included asthma, cystic fibrosis, arthritis, diabetes, lupus and epilepsy. Two hundred and forty seven of the 437 adolescents invited to participate returned the ARQ (57% response rate). Forty three respondents were ineligible as they were over 18 years of age, which left a total of 204 adolescents with a mean age of 14.9 years (see Table 1).

#### Analysis

Seven items commonly identified as difficult to understand and seven items with endorsement of less the 20% or greater than 80% were deleted. Factor solutions were found to be almost identical in the chronic illness and school samples so the data were combined to increase sample size (n = 534). Factors closely reflected the conceptually developed scales and explained the majority of the variance in all solutions with the exception of the individual and community domains, where the most revision occurred (data available in additional files as detailed below).

In the individual domain the six factor scales resembled the six conceptually derived scales (see additional file 1 for factor output for the individual domain), however a number of important differences were identified and acted upon (see Figure 1). The *emotion regulation *factor scale appeared more focussed on negative cognition, due in part to the positive *emotion regulation *items loading on the *optimism/positive future expectation *factor scale. Therefore, it was decided to:

• Rename the *emotion regulation *factor scale *negative cognition *and add new items to cover negative emotion regulation more comprehensively.

• Add new items to the *optimism/positive future expectation *scale to more closely reflect the construct.

• Expand the *introspection/meaning *scale to include items addressing positive emotion regulation and rename the scale *introspection/emotional insight*, as these items are conceptually a better fit here than in the *optimism/positive future expectation *factor scale.

New items were added accordingly and a number of items were rewritten in the remaining scales so that items reflected the underlying construct more closely or to simplify the language. As described in the method section above, new items were written following the guiding principals elaborated by Kline [57] and Streiner and Norman [58], drawing on the conceptual underpinning of the scale and taking into account items that had failed to perform. In the family, peer and school domains, the conceptual scale structure of *connectedness *and *availability of support *was replicated by the factor analysis (see additional files 2, 3, 4 for factor output for family, peer and school domains). There was some movement of individual items between scales and some items were rewritten to be more specific and less positive in order to improve the face and content validity. In the community domain the two conceptually developed scales were supported by the factor structure (see additional file 5 for factor output for community domain), but were highly correlated (r = 0.7). Revision of these scales therefore focused on developing scales that tapped more discrete constructs by drawing on the sense of community and social capital literature (see [59-61]). The *connectedness *scale already covered *sense of community*, addressing adolescents' perception of belonging and attachment to their neighbourhood. Therefore the *support availability *scale was broadened with the addition of new items aimed at encompassing social capital concepts of trust, obligation and sanction [61].

Following this process, the pilot ARQ was revised to create the ARQ-Revision 1 (ARQ-Rev1) which was comprised of six scales and 79 items in the individual domain, and two scales each in the family (20 items), peer (11 items), school (15 items) and community (15 items) domains. Whilst the ARQ-Rev1 was relatively long (140 items in total), being over inclusive at this stage of development facilitated selection of the best items and scales in the second phase of data collection and revision described in Study 2.

### Study 2 - Revision of the ARQ-Rev1 to create a brief functional measure of resilience

#### Participants

Eleven of 12 secondary schools randomly selected from all Victorian Government schools agreed to participate in the study. Two classes from years seven and nine were randomly selected within each school^1^. A total of 451 of 982 eligible students (50% response rate) completed the questionnaire during class time. Students had a mean age of 13.9 years (see Table 1).

#### Analysis

Two items commonly seen as difficult to understand and nine items with endorsement of less than 20% or more than 80% were deleted.

In the individual domain, the 5-factor solution was selected as the most parsimonious as described in the above methods section (see additional file 6 for factor output for the individual domain). Major similarities and differences were:

• Four factors closely resembled the ARQ-Rev1 scales of *negative cognition, empathy, social skills *and *emotional insight/introspection *and were labelled accordingly.

• Items from the ARQ-Rev1 *self-confidence *and *optimism/positive future expectation *scales loaded on a single factor. This factor was therefore labelled *confidence (self and future) *(see Figure 1).

• The ARQ-Rev1 *problem solving *scale was not supported by the factor analysis, with problem solving items loading on various factors in the solution.

The five factors were used to construct scales following the steps described in the method. Following this revision process the individual domain consisted of the five scales *negative cognition*, *confidence (self and future)*, *emotional insight*, *empathy/tolerance *and *social skills*. The scales contained six to eight items and, as shown in Table 2, scale reliabilities ranged from adequate to very good. Two new items were added to the *empathy *and *social skills *scales with the intention of improving scale reliability. The items were written as described in the method section above, drawing on the conceptual underpinning of the scale and taking into account items that had failed to perform in the previous two revisions.

**Table 2:** ARQ scales, example item and reliability score

DOMAIN Scale	Sample item	Number of items	Reliability (Cronbach α)
INDIVIDUAL			
Confidence (self/future)	I feel confident that I can handle whatever comes my way	8	0.8
Emotional insight	I think things through carefully before making decisions	8	0.7
Negative cognition	I tend to think the worst is going to happen (reversed)	8	0.8
Social skills	I can express my opinions when I am in a group	8	0.7
Empathy/Tolerance	I am patient with people who can't do things as well as I can	8	0.7
			
FAMILY			
Connectedness	I enjoy spending time with my family	8	0.9
Availability	There is someone in my family I can talk to about anything	3	0.8
PEERS			
Connectedness	I have a friend I can trust with my private thoughts and feelings	7	0.8
Availability	I wish I had more friends I felt close to (reversed)	8	0.6
SCHOOL			
Supportive Environment	My teachers are caring and supportive of me	8	0.8
Connectedness	I try hard in school	8	0.7
COMMUNITY			
Connectedness	I trust the people in my neighbourhood	6	0.9

In the family domain, the two factor solution closely replicated the ARQ-Rev1 scales of *connectedness *and *availability *and was labelled accordingly (see additional file 7 for factor output for the family domain). The two factors were used to construct scales with excellent reliability (see Table 2). The *connectedness *scale assesses a nurturing and supportive family environment, while the second scale assesses the *availability *of family members for support or advice. The two family scales were highly correlated (r = 0.66) indicating that adolescents' scores on family *connectedness *will generally correspond to their scores on the *availability *scale. Similarly, the factor analysis in the peer domain closely replicated the ARQ-Rev1 peer *connectedness *and *availability *scales (see additional file 8 for factor output for the peer domain). The *connectedness *scale covers feeling connected to friends and confidence with peers, while the *availability *scale (reversed) taps into the ability to form and maintain friendships. The *connectedness *scale had excellent reliability; however, the reliability of the three item *availability *scale fell below the target range of 0.7 - 0.9. Therefore five new items were written to create an eight-item scale with the intention of improving reliability.

The factor analysis in the school domain closely replicated the ARQ-Rev1 *supportive environment *and *connectedness *scales (see additional file 9 for factor output for the school domain). Items in the *supportive environment *scale refer to student and staff factors that impact on the general school environment. The *connectedness *scale (reversed) contains items related to an adolescents' feelings of commitment and connection to school both social and academically. Four new items were added to the connectedness scale to make an eight-item scale, with the intention of balancing the scale with positive items and improving the reliability coefficient.

Efforts to address the multiple facets of community support and belonging as explored in the sense of community and social capital literature failed to be supported by the factor analysis in the *community domain*. The data consistently identified a single factor, seemingly addressing general community connectedness (see additional file 10 for factor output for the community domain). The eight item community scale had a Cronbach alpha coefficient greater than 0.90, suggesting excessive consistency or repetitive items in the scale [62]. Two items were therefore deleted to produce a six-item scale with excellent reliability (see Table 2). The community connectedness scale assesses networks of support and engagement within the community.

The revised ARQ comprises six scales in the individual domain, two scales in each of the family, peer, and school domains and a single community scale with 88 items in total.

## Discussion

While the literature around the concept of resilience is increasing, there have been few attempts to synthesise current research findings into useful measurement tools. The newly developed ARQ is a relatively short and easy to administer questionnaire which identifies the resources available to an adolescent, both individually and in their wider social environment. The ARQ can assist in identifying adolescents who have personal characteristics associated with resilience (confidence, social skills, emotional insight and negative cognition) and who are positively engaged with their family, peers, school and community environments. Arguably, such adolescents are more likely to show resilient outcomes in times of adversity. Conversely, the ARQ can identify adolescents who show poor engagement in all or some of these areas and who may be vulnerable in the face of adversity.

The ARQ scales identified in the factor analyses show notable conceptual similarity to the constructs initially proposed for measurement (drawn from the resilience literature and focus groups). However some scales were not supported by the statistical analysis. For example, the *problem solving *scale failed to form a unique factor, with items loading on the *social skills *and *emotional insight *factors, and was ultimately subsumed into these two scales. This may reflect poor construct operationalisation or be a true reflection of the adolescent experience. Resilience literature supports the latter, reporting significant positive associations between problem solving skills and both social skills and emotion regulation in childhood, adolescence and adulthood [31,33,63-65]. Thus, problem solving skills may not operate as a singular competency but underlie other resilience factors, such as emotional insight and social skills, and be context specific.

The identification of the *negative cognition *factor was unexpected and without precedence in other resilience measures. Many of the *negative cognition *items were negatively worded and were intended to gauge deficits in self efficacy, confidence and optimism/hope. Few resilience measures have included negative items as they generally assess possession of a resource rather than a deficit. This factor appears to address a sense of helplessness and low internal locus of control. Children identified in the Rochester Child Resilience Project as stress-affected [36] evidenced such characteristics - they had significantly lower scores than both stress-resilient children and non-classified children on problem solving, coping and internal locus of control. Thus, conceptually, the *negative cognition *scale defines vulnerable adolescents, such that high scores on this scale would be expected to be associated with low resilience. Further testing and comparison with other measures of resilience will add to the understanding of this scale.

The two family scales were highly correlated indicating that adolescents' scores on family *connectedness *will generally correspond to their scores on the *availability *scale. It may therefore be preferable to use only one of these two scales. However, the items loaded cleanly on separate factors in the analysis, suggesting the scales tapped into different constructs. Further investigation, including tests of construct validity, will allow an informed decision to be made as to whether to retain both scales or to retain one scale and decrease of the length of the measure - a desirable goal in scale development.

The ARQ is at an early stage of development and further psychometric testing is necessary. For example, the comparison of ARQ scores and relevant sub-scales with other resilience measures, and comparison of sub-scales with gold standard measures of similar concepts such as coping and social support will provide evidence of criterion validity. Examination of ARQ scores for identified stress-resilient and stress-affected populations will indicate how well the ARQ discriminates between populations. Focus groups and administration of the questionnaire to over 500 adolescents in total have shown the questionnaire to be easy for adolescents to understand and complete. The factor and scale analyses revealed a stable factor structure that was conceptually convincing and true to the original intent of the measure. While the ARQ is presented as a functional measure of resilience in adolescents, evidence of scale and test-retest reliability, criterion validity and sensitivity to change will enhance understanding of and confidence in the measure's psychometric properties.

The ARQ has been developed to identify adolescents who have personal characteristics associated with resilience, and who are positively engaged with their family, peers, school and community environments. Such adolescents are more likely to show resilient outcomes in times of adversity. Conversely, the ARQ can identify adolescents who show deficits or poor engagement in all or some of these areas, who may be vulnerable in the face of adversity. The availability of developmentally appropriate, multidimensional measurement tools will facilitate resilience researchers in their 'central mission' as described by Luthar and Brown "... to illuminate processes that significantly mitigate the ill effects of various adverse life conditions as well as those that exacerbate these, and thus to derive specific directions for interventions and social policies" [66].

## Conclusions

The ARQ was developed in response to a distinct lack of measurement tools in resilience research generally and for adolescents in particular. Greater scientific rigour and consistency in measurement tools and approaches will contribute to improved understanding of the complex processes involved in resilient responses to adversity. The availability of standard measures in resilience research, such as the ARQ, will make comparisons across studies and risk groups possible. With further psychometric testing, this new measure of resilience will provide researchers and clinicians with a comprehensive and developmentally appropriate instrument to measure a young person's *capacity *to achieve positive outcomes despite life stressors.

## Competing interests

The authors declare that they have no competing interests.

## Authors' contributions

DG participated in the design of the studies, coordinated and carried out data collection, performed the statistical analysis and drafted the manuscript. LB participated in the design of the studies, advised on data analysis and revised the manuscript. CO conceived of the study, participated in design, led the focus groups and revised the manuscript. SB participated in the design of the studies, was the principal supervisor of the doctoral thesis upon which this article was based and revised the manuscript. SS participated in the design of the study and revised the manuscript. All authors read and approved the final manuscript.

## Endnotes

^1^With the exception of one rural school where an unforeseen school event determined that year 7 classes were unavailable at the prearranged time and all year nine students were surveyed instead. We were unable to arrange another time due to school commitments and ethics requirements prohibited the substitution of another year level.

## Pre-publication history

The pre-publication history for this paper can be accessed here:

http://www.biomedcentral.com/1471-2288/11/134/prepub

## Supplementary Material

Additional file 1 **Study 1 Factor solution individual domain**. Study 1 output describing factor analysis of the individual domain. Output includes the initial statistics for the six-factor solution with oblimin rotation, and the rotated factor loadings with the original conceptual scales, and factor developed scales described. Click here for file

Additional file 2 **Study 1 Factor solution family domain**. Study 1 output describing factor analysis of the family domain. Output includes the initial statistics for the two-factor solution with oblimin rotation, and the rotated factor loadings with the original conceptual scales, and factor developed scales described. Click here for file

Additional file 3 **Study 1 Factor solution peer domain**. Study 1 output describing factor analysis of the peer domain. Output includes the initial statistics for the two-factor solution with oblimin rotation, and the rotated factor loadings with the original conceptual scales, and factor developed scales described. Click here for file

Additional file 4 **Study 1 Factor solution school domain**. Study 1 output describing factor analysis of the school domain. Output includes the initial statistics for the two-factor solution with oblimin rotation, and the rotated factor loadings with the original conceptual scales, and factor developed scales described. Click here for file

Additional file 5 **Study 1 Factor solution community domain**. Study 1 output describing factor analysis of the community domain. Output includes the initial statistics for the two-factor solution with oblimin rotation, and the rotated factor loadings with the original conceptual scales, and factor developed scales described. Click here for file

Additional file 6 **Study 2 Factor solution individual domain**. Study 2 output describing factor analysis of the individual domain. Output includes the initial statistics for the five-factor solution with oblimin rotation, and the rotated factor loadings with the original conceptual scales, and factor developed scales described. Click here for file

Additional file 7 **Study 2 Factor solution family domain**. Study 2 output describing factor analysis of the family domain. Output includes the initial statistics for the two-factor solution with oblimin rotation, and the rotated factor loadings with the original conceptual scales, and factor developed scales described. Click here for file

Additional file 8 **Study 2 Factor solution peer domain**. Study 2 output describing factor analysis of the peer domain. Output includes the initial statistics for the two-factor solution with oblimin rotation, and the rotated factor loadings with the original conceptual scales, and factor developed scales described. Click here for file

Additional file 9 **Study 2 Factor solution school domain**. Study 2 output describing factor analysis of the school domain. Output includes the initial statistics for the two-factor solution with oblimin rotation, and the rotated factor loadings with the original conceptual scales, and factor developed scales described. Click here for file

Additional file 10 **Study 2 Factor solution community domain**. Study 2 output describing factor analysis of the community domain. Output includes the initial statistics for the two-factor solution with oblimin rotation, and the rotated factor loadings with the original conceptual scales, and factor developed scales described. Click here for file
