# Supplementary material for: Development of a multi-dimensional measure of resilience in adolescents: the Adolescent Resilience Questionnaire
Source: BMC Med Res Methodol. 2011 Oct 5;11:134. doi: 10.1186/1471-2288-11-134 (PMC3204306; doi:10.1186/1471-2288-11-134)
Supplement: Additional file 1 — Study 1 Factor solution individual domain. Study 1 output describing factor analysis of the individual domain. Output includes the initial statistics for the six-factor solution with oblimin rotation, and the rotated factor loadings with the original conceptual scales, and factor developed scales described. [file 1471-2288-11-134-S1.DOCX]

**Additional file 1. Study 1 Factor output for the individual domain**

Initial statistics for a six-factor solution with oblimin rotation (n=534)

| Total Variance Explained | | | | |
| --- | --- | --- | --- | --- |
| Factor | Initial Eigenvalues | | | Rotation Sums of Squared Loadings^a^ |
|  | Total | % of Variance | Cumulative % | Total |
| 1 | 9.09 | 18.94 | 18.94 | 6.52 |
| 2 | 4.25 | 8.86 | 27.80 | 2.57 |
| 3 | 2.34 | 4.88 | 32.68 | 3.35 |
| 4 | 1.69 | 3.53 | 36.21 | 4.53 |
| 5 | 1.58 | 3.28 | 39.49 | 4.23 |
| 6 | 1.43 | 2.99 | 42.48 | 3.16 |
| 7 | 1.22 | 2.53 | 45.01 |  |
| 8 | 1.18 | 2.45 | 47.46 |  |
| 9 | 1.14 | 2.38 | 49.84 |  |
| 10 | 1.09 | 2.27 | 52.11 |  |
| 11 | 1.07 | 2.23 | 54.33 |  |
| 12 | 1.06 | 2.20 | 56.53 |  |
| 13 | 0.96 | 2.00 | 58.54 |  |
| 14 | 0.91 | 1.90 | 60.43 |  |
| 15 | 0.90 | 1.88 | 62.32 |  |
| 16 | 0.84 | 1.75 | 64.06 |  |
| 17 | 0.81 | 1.69 | 65.76 |  |
| 18 | 0.79 | 1.65 | 67.41 |  |
| 19 | 0.78 | 1.61 | 69.02 |  |
| 20 | 0.76 | 1.57 | 70.60 |  |
| 21 | 0.74 | 1.54 | 72.13 |  |
| 22 | 0.72 | 1.50 | 73.63 |  |
| 23 | 0.70 | 1.47 | 75.10 |  |
| 24 | 0.69 | 1.43 | 76.53 |  |
| 25 | 0.67 | 1.40 | 77.93 |  |
| 26 | 0.64 | 1.34 | 79.27 |  |
| 27 | 0.64 | 1.33 | 80.60 |  |
| 28 | 0.62 | 1.30 | 81.90 |  |
| 29 | 0.58 | 1.21 | 83.11 |  |
| 30 | 0.57 | 1.19 | 84.30 |  |
| 31 | 0.56 | 1.17 | 85.47 |  |
| 32 | 0.55 | 1.15 | 86.62 |  |
| 33 | 0.52 | 1.09 | 87.70 |  |

Initial statistics for the individual domain (Continued)

| Total Variance Explained | | | | |
| --- | --- | --- | --- | --- |
| Factor | Initial Eigenvalues | | | Rotation Sums of Squared Loadings^a^ |
|  | Total | % of Variance | Cumulative % | Total |
| 34 | 0.50 | 1.04 | 88.74 |  |
| 35 | 0.49 | 1.02 | 89.76 |  |
| 36 | 0.48 | 1.00 | 90.77 |  |
| 37 | 0.45 | 0.95 | 91.71 |  |
| 38 | 0.44 | 0.91 | 92.62 |  |
| 39 | 0.43 | 0.89 | 93.51 |  |
| 40 | 0.41 | 0.85 | 94.37 |  |
| 41 | 0.40 | 0.84 | 95.20 |  |
| 42 | 0.37 | 0.78 | 95.98 |  |
| 43 | 0.36 | 0.74 | 96.72 |  |
| 44 | 0.35 | 0.74 | 97.46 |  |
| 45 | 0.34 | 0.71 | 98.17 |  |
| 46 | 0.32 | 0.67 | 98.84 |  |
| 47 | 0.29 | 0.60 | 99.44 |  |
| 48 | 0.27 | 0.56 | 100.00 |  |
| Extraction Method: Maximum Likelihood. | | | | |
| a. When factors are correlated, sums of squared loadings cannot be added to obtain a total variance. | | | | |

Factor solution for the individual domain (n=534)

| Conceptual Scale^a^ | Factor Scale^b^ | 1 | 2 | 3 | 4 | 5 | 6 |
| --- | --- | --- | --- | --- | --- | --- | --- |
|  | **Emotional insight (negative)** |  |  |  |  |  |  |
| Optimism | I tend to think the worst is going to happen | 0.61 |  |  |  |  |  |
| Emotion | I find it difficult to cope when things change unexpectedly | 0.60 |  |  |  |  |  |
| Problem | I feel helpless when faced with a problem | 0.60 |  |  |  |  |  |
| Problem | If one approach to a problem doesn’t work, I find it hard to think of other ideas | 0.52 |  |  |  |  |  |
| Emotion | If something upsets me it affects how I feel about everything | 0.51 |  |  |  |  |  |
| Emotion | When things go wrong I give myself a hard time | 0.50 |  |  |  |  |  |
| Emotion | I feel that I have little control | 0.50 |  |  |  |  |  |
| Communication | I have trouble explaining how I am feeling | 0.49 |  |  |  |  |  |
| Emotion | When I make a mistake I feel that I am a hopeless person | 0.47 |  |  |  |  |  |
| Empathy | I easily get frustrated with people | 0.46 |  |  |  |  |  |
| Optimism | I worry about the future | 0.43 |  |  |  |  |  |
| Emotion | I am not happy unless things are perfect | 0.41 |  |  |  |  |  |
| Emotion | I push myself too hard to do what everyone else does | 0.40 |  |  |  |  |  |
| Emotion | I worry about what people are thinking about me | 0.39 |  |  |  |  |  |
| Communication | I find it hard to express myself to others | 0.35 |  | 0.32 |  |  |  |
| Problem | If something is becoming a problem I try to ignore it | 0.31 |  |  |  |  |  |
| Emotion | When people say nice things about me |  |  |  |  |  |  |
|  | **Introspection/Meaning** |  |  |  |  |  |  |
| Introspection | I like to think about why things happen the way they do |  | 0.62 |  |  |  |  |
| Introspection | I try to find meaning in the things that happen to me |  | 0.56 |  |  |  |  |
| Empathy | I think about what things might be like for other people |  | 0.46 |  |  |  |  |
| Introspection | Even if it isn’t clear to me I believe things happen for a reason |  | 0.40 |  |  |  |  |
|  | **Problem Solving/Help seeking** |  |  |  |  |  |  |
| Problem | I keep my problems to myself |  |  | 0.74 |  |  |  |
| Problem | If I have a problem I deal with it by myself |  |  | 0.64 |  |  |  |
| Communication | I am a private person when it comes to how I feel |  |  | 0.59 |  |  |  |
| Problem | If I have a problem, I know there is someone I can talk to |  |  | -0.47 |  |  |  |
| Problem | If I cant handle something I find help |  |  | -0.46 | 0.54 |  |  |
|  | **Optimism/Hope** |  |  |  |  |  |  |
| Optimism | I try to take a relaxed approach to things |  |  |  | 0.52 |  |  |
| Emotion | If I get upset, I know how to make myself feel better |  |  |  | 0.49 |  |  |
| Optimism | I try to make the best out of situations |  |  |  | 0.48 |  |  |
| Optimism | I feel hopeful about my life |  |  |  | 0.45 |  |  |
| Optimism | Seeing the funny side of situations helps me when things get bad |  |  |  | 0.38 |  |  |
| Emotion | I understand why I feel the way I do |  |  |  | 0.36 |  |  |
| Introspection | I look for what I can learn from bad things that happen |  |  |  | 0.32 |  |  |
| Optimism | I take it easy on myself when I am not feeling well |  |  |  |  |  |  |
|  | **Social skills** |  |  |  |  |  |  |
| Agreeableness | I make friends easily |  |  |  |  | 0.66 |  |
| Agreeableness | I have a hard time getting along with others |  |  |  |  | -0.57 |  |
| Agreeableness | I enjoy meeting new people |  |  |  |  | 0.43 |  |
| Communication | I find it easy to talk to people |  |  | -0.36 | 0.31 | 0.42 |  |
| Problem | I can stand up for myself when there is a problem |  |  |  |  | 0.33 |  |
| Agreeableness | I enjoy spending time by myself |  |  |  |  | -0.30 |  |
| Optimism | I feel confident that I will have a romantic relationship |  |  |  |  |  |  |

Factor solution for the individual domain (continued).

| Conceptual Scale^a^ | Factor Scale | 1 | 2 | 3 | 4 | 5 | 6 |
| --- | --- | --- | --- | --- | --- | --- | --- |
|  | **Empathy** |  |  |  |  |  |  |
| Empathy | I am a good listener |  |  |  |  |  | 0.72 |
| Empathy | I listen carefully to my friends when they have problems |  |  |  |  |  | 0.62 |
| Empathy | People who know me think that I am understanding |  |  |  |  |  | 0.46 |
| Optimism | I try to live a healthy life |  |  |  |  |  | 0.30 |
| Problem | I carefully consider all options before making decisions |  |  |  |  |  | 0.30 |
| Empathy | I feel obliged to do the right thing by others |  |  |  |  |  |  |
| Empathy | I am forgiving of other people |  |  |  |  |  |  |

a. Column one identifies the conceptual scale each item was associated with.

b. Maximum Likelihood extraction and Oblimin rotation with Kaiser normalisation.
