# Supplementary material for: Development of a multi-dimensional measure of resilience in adolescents: the Adolescent Resilience Questionnaire
Source: BMC Med Res Methodol. 2011 Oct 5;11:134. doi: 10.1186/1471-2288-11-134 (PMC3204306; doi:10.1186/1471-2288-11-134)
Supplement: Additional file 2 — Study 1 Factor solution family domain. Study 1 output describing factor analysis of the family domain. Output includes the initial statistics for the two-factor solution with oblimin rotation, and the rotated factor loadings with the original conceptual scales, and factor developed scales described. [file 1471-2288-11-134-S2.DOCX]

**Additional file 2. Study 1: Factor output for family domain**

Initial statistics for a two-factor solution with oblimin rotation (n=534)

| Total Variance Explained | | | | |
| --- | --- | --- | --- | --- |
| Factor | Initial Eigenvalues | | | Rotation Sums of Squared Loadings^a^ |
|  | Total | % of Variance | Cumulative % | Total |
| 1 | 6.38 | 49.04 | 49.04 | 5.76 |
| 2 | 1.12 | 8.59 | 57.63 | 3.04 |
| 3 | 0.90 | 6.93 | 64.56 |  |
| 4 | 0.87 | 6.70 | 71.26 |  |
| 5 | 0.68 | 5.20 | 76.47 |  |
| 6 | 0.65 | 4.98 | 81.45 |  |
| 7 | 0.57 | 4.35 | 85.80 |  |
| 8 | 0.43 | 3.32 | 89.11 |  |
| 9 | 0.35 | 2.67 | 91.78 |  |
| 10 | 0.33 | 2.51 | 94.29 |  |
| 11 | 0.27 | 2.10 | 96.39 |  |
| 12 | 0.25 | 1.94 | 98.34 |  |
| 13 | 0.22 | 1.66 | 100.00 |  |
| Extraction Method: Maximum Likelihood. | | | | |

a. When factors are correlated, sums of squared loadings cannot be added to obtain a total variance.

Factor solution for the family domain (n = 534)

| Conceptual scale | Factor | 1 | 2 |
| --- | --- | --- | --- |
|  | **Connectedness** |  |  |
| Connectedness | My family understands my needs | 0.84 |  |
| Connectedness | My family is caring | 0.79 |  |
| Communication | My family listens to me | 0.78 |  |
| Connectedness | I feel close to my family | 0.77 |  |
| Availability | My family is there for me when I need them | 0.73 |  |
| Communication | I can be honest with my family about how I feel | 0.69 |  |
| Connectedness | I enjoy spending time with my family | 0.66 |  |
| Connectedness | My family puts me down | -0.60 |  |
| Communication | I have a say in family decisions | 0.58 |  |
| Connectedness | My family is over protective of me | < 0.30 |  |
| Availability | The amount of time I spend doing things with my family is: (Too little/Enough/Too much) | < 0.30 |  |
|  | **Availability** |  |  |
| Availability | If I have a problem there is someone in my family I can talk to |  | -0.91 |
| Availability | There is someone in my family that I feel very close to |  | -0.50 |

a. Column one identifies the conceptual scale each item was associated with.

b. Maximum Likelihood extraction and Oblimin rotation with Kaiser normalisation.
