# Supplementary material for: Development of a multi-dimensional measure of resilience in adolescents: the Adolescent Resilience Questionnaire
Source: BMC Med Res Methodol. 2011 Oct 5;11:134. doi: 10.1186/1471-2288-11-134 (PMC3204306; doi:10.1186/1471-2288-11-134)
Supplement: Additional file 3 — Study 1 Factor solution peer domain. Study 1 output describing factor analysis of the peer domain. Output includes the initial statistics for the two-factor solution with oblimin rotation, and the rotated factor loadings with the original conceptual scales, and factor developed scales described. [file 1471-2288-11-134-S3.DOCX]

**Additional file 3. Study 1. Factor output for peer domain**

Initial statistics for a two-factor solution with oblimin rotation (n=534)

| Total Variance Explained | | | | |
| --- | --- | --- | --- | --- |
| Factor | Initial Eigenvalues | | | Rotation Sums of Squared Loadings^a^ |
|  | Total | % of Variance | Cumulative % | Total |
| 1 | 3.81 | 47.57 | 47.57 | 3.02 |
| 2 | 1.04 | 12.95 | 60.52 | 2.60 |
| 3 | 0.83 | 10.41 | 70.93 |  |
| 4 | 0.62 | 7.79 | 78.73 |  |
| 5 | 0.59 | 7.35 | 86.08 |  |
| 6 | 0.49 | 6.13 | 92.21 |  |
| 7 | 0.37 | 4.62 | 96.83 |  |
| 8 | 0.25 | 3.17 | 100.00 |  |
| Extraction Method: Maximum Likelihood. | | | | |
| a. When factors are correlated, sums of squared loadings cannot be added to obtain a total variance. | | | | |

Factor solution for the peer domain (n = 534)

| Conceptual scale | ARQ-Pilot | 1 | 2 |
| --- | --- | --- | --- |
|  | **Connectedness** |  |  |
| Connectedness | I have fun with my friends | 0.80 | 0.30 |
| Connectedness | My friends like doing the same things as me | 0.71 |  |
| Connectedness | My friends leave me out of things | -0.64 |  |
| Availability | I have a group of friends that I keep in touch with | 0.56 | 0.42 |
| Connectedness | My friends are caring and supportive | 0.47 |  |
| Availability | The amount of time I spend with my friend(s) is | -0.34 |  |
|  | (Too little/enough/Too much) |  |  |
|  | **Availability** |  |  |
| Availability | I have a friend(s) that I feel close to |  | -0.89 |
| Availability | I have a friend(s) that I can talk to about anything |  | -0.80 |

a. Column one identifies the conceptual scale each item was associated with.

b. Maximum Likelihood extraction and Oblimin rotation with Kaiser normalisation.
