# Supplementary material for: Development of a multi-dimensional measure of resilience in adolescents: the Adolescent Resilience Questionnaire
Source: BMC Med Res Methodol. 2011 Oct 5;11:134. doi: 10.1186/1471-2288-11-134 (PMC3204306; doi:10.1186/1471-2288-11-134)
Supplement: Additional file 4 — Study 1 Factor solution school domain. Study 1 output describing factor analysis of the school domain. Output includes the initial statistics for the two-factor solution with oblimin rotation, and the rotated factor loadings with the original conceptual scales, and factor developed scales described. [file 1471-2288-11-134-S4.DOCX]

**Additional file 4. Study 1 Factor output for school domain**

Initial statistics for a two-factor solution with oblimin rotation (n=534)

| Total Variance Explained | | | | |
| --- | --- | --- | --- | --- |
| Factor | Initial Eigenvalues | | | Rotation Sums of Squared Loadings^a^ |
|  | Total | % of Variance | Cumulative % | Total |
| 1 | 3.65 | 33.18 | 33.18 | 2.79 |
| 2 | 1.53 | 13.88 | 47.05 | 2.07 |
| 3 | 1.08 | 9.85 | 56.91 |  |
| 4 | 0.87 | 7.90 | 64.81 |  |
| 5 | 0.76 | 6.91 | 71.72 |  |
| 6 | 0.76 | 6.90 | 78.62 |  |
| 7 | 0.64 | 5.78 | 84.40 |  |
| 8 | 0.49 | 4.46 | 88.87 |  |
| 9 | 0.48 | 4.33 | 93.20 |  |
| 10 | 0.42 | 3.84 | 97.04 |  |
| 11 | 0.33 | 2.96 | 100.00 |  |
| Extraction Method: Maximum Likelihood. | | | | |

a. When factors are correlated, sums of squared loadings cannot be added to obtain a total variance.

Factor solution for the school domain (n = 534)

| Conceptual scale | ARQ-Pilot | 1 | 2 |
| --- | --- | --- | --- |
|  | **Support** |  |  |
| Environment | My teachers are caring and supportive | 0.90 |  |
| Environment | I have a teacher that I feel looks out for me | 0.73 |  |
| Environment | My teachers provide me with extra help if I need it | 0.67 |  |
| Environment | My teachers expect too much of me | -0.43 |  |
| Environment | There is an adult at school that I could talk to if I had a personal problem | 0.35 |  |
| Connected | Doing well at school is important to me | <0.30 |  |
|  | **Connectedness** |  |  |
| Connected | I feel left out at school |  | 0.66 |
| Connected | I get bullied or teased at school |  | 0.64 |
| Environment | I feel safe at school |  | -0.61 |
| Connected | I hate going to school |  | 0.33 |
| Connected | I get involved with school activities |  | -0.30 |

a. Column one identifies the conceptual scale each item was associated with.

b. Maximum Likelihood extraction and Oblimin rotation with Kaiser normalisation.
