# Supplementary material for: Development of a multi-dimensional measure of resilience in adolescents: the Adolescent Resilience Questionnaire
Source: BMC Med Res Methodol. 2011 Oct 5;11:134. doi: 10.1186/1471-2288-11-134 (PMC3204306; doi:10.1186/1471-2288-11-134)
Supplement: Additional file 5 — Study 1 Factor solution community domain. Study 1 output describing factor analysis of the community domain. Output includes the initial statistics for the two-factor solution with oblimin rotation, and the rotated factor loadings with the original conceptual scales, and factor developed scales described. [file 1471-2288-11-134-S5.DOCX]

**Additional file 5. Study 1 Factor output for community domain**

Initial statistics for a two-factor solution with oblimin rotation (n=534)

| Total Variance Explained | | | | |
| --- | --- | --- | --- | --- |
| Factor | Initial Eigenvalues | | | Rotation Sums of Squared Loadings^a^ |
|  | Total | % of Variance | Cumulative % | Total |
| 1 | 3.76 | 46.99 | 46.99 | 3.02 |
| 2 | 1.04 | 12.99 | 59.98 | 2.76 |
| 3 | 0.92 | 11.47 | 71.45 |  |
| 4 | 0.71 | 8.82 | 80.27 |  |
| 5 | 0.62 | 7.80 | 88.06 |  |
| 6 | 0.41 | 5.11 | 93.18 |  |
| 7 | 0.28 | 3.52 | 96.70 |  |
| 8 | 0.26 | 3.30 | 100.00 |  |
| Extraction Method: Maximum Likelihood. | | | | |

a. When factors are correlated, sums of squared loadings cannot be added to obtain a total variance.

Factor solution for the community domain (n = 534)

| Conceptual scale | ARQ-Pilot | 1 | 2 |
| --- | --- | --- | --- |
|  | **Connectedness** |  |  |
| Connectedness | I like my neighbourhood | 0.85 |  |
| Connectedness | I like the people in my neighbourhood | 0.77 |  |
| Connectedness | I feel isolated in my neighbourhood | 0.53 |  |
| Connectedness | I trust the people in my neighbourhood | -0.41 |  |
|  | **Availability** |  |  |
| Availability | People in my neighbourhood go out of their way to help |  | 0.91 |
| Availability | People in my neighbourhood are caring |  | 0.58 |
| Availability | There is an adult in my neighbourhood that I … |  | 0.35 |
| Connectedness | I am part of a social group in my neighbourhood which is not run by my school |  | <0.30 |

a. Column one identifies the conceptual scale each item was associated with.

b. Maximum Likelihood extraction and Oblimin rotation with Kaiser normalisation.
