# Supplementary material for: Development of a multi-dimensional measure of resilience in adolescents: the Adolescent Resilience Questionnaire
Source: BMC Med Res Methodol. 2011 Oct 5;11:134. doi: 10.1186/1471-2288-11-134 (PMC3204306; doi:10.1186/1471-2288-11-134)
Supplement: Additional file 6 — Study 2 Factor solution individual domain. Study 2 output describing factor analysis of the individual domain. Output includes the initial statistics for the five-factor solution with oblimin rotation, and the rotated factor loadings with the original conceptual scales, and factor developed scales described. [file 1471-2288-11-134-S6.DOCX]

**Additional file 6. Study 2** **Factor output for the individual domain**

Initial statistics for a 5-factor solution with oblimin rotation (n = 451)

| Factor | Initial Eigenvalues | | | Rotation Sums of Squared Loadings(a) |
| --- | --- | --- | --- | --- |
|  | Total | % of Variance | Cumulative % | Total |
| 1 | 10.74 | 15.34 | 15.34 | 6.98 |
| 2 | 5.97 | 8.52 | 23.86 | 6.69 |
| 3 | 2.49 | 3.56 | 27.42 | 3.76 |
| 4 | 2.09 | 2.99 | 30.41 | 4.58 |
| 5 | 1.89 | 2.70 | 33.11 | 4.41 |
| 6 | 1.80 | 2.57 | 35.68 |  |
| 7 | 1.67 | 2.39 | 38.07 |  |
| 8 | 1.45 | 2.07 | 40.15 |  |
| 9 | 1.38 | 1.97 | 42.11 |  |
| 10 | 1.34 | 1.92 | 44.03 |  |
| 11 | 1.32 | 1.88 | 45.92 |  |
| 12 | 1.28 | 1.82 | 47.74 |  |
| 13 | 1.23 | 1.76 | 49.51 |  |
| 14 | 1.19 | 1.70 | 51.21 |  |
| 15 | 1.15 | 1.64 | 52.85 |  |
| 16 | 1.12 | 1.60 | 54.45 |  |
| 17 | 1.08 | 1.55 | 56.00 |  |
| 18 | 1.04 | 1.49 | 57.49 |  |
| 19 | 1.01 | 1.44 | 58.93 |  |
| 20 | 1.00 | 1.43 | 60.37 |  |
| 21 | 0.98 | 1.40 | 61.77 |  |
| 22 | 0.96 | 1.37 | 63.13 |  |
| 23 | 0.93 | 1.33 | 64.47 |  |
| 24 | 0.92 | 1.32 | 65.78 |  |
| 25 | 0.90 | 1.29 | 67.08 |  |
| 26 | 0.90 | 1.28 | 68.36 |  |
| 27 | 0.84 | 1.19 | 69.55 |  |
| 28 | 0.82 | 1.17 | 70.72 |  |
| 29 | 0.80 | 1.14 | 71.86 |  |
| 30 | 0.76 | 1.09 | 72.95 |  |
| 31 | 0.75 | 1.07 | 74.02 |  |
| 32 | 0.74 | 1.05 | 75.07 |  |
| 33 | 0.73 | 1.04 | 76.11 |  |
| 34 | 0.70 | 1.00 | 77.11 |  |
| 35 | 0.69 | 0.98 | 78.09 |  |
| 36 | 0.66 | 0.94 | 79.03 |  |
|  |  |  |  |  |

Factor solution for the individual domain (n = 451)

| ARQ-Rev1 Scale | Factor | Factor | | | | |
| --- | --- | --- | --- | --- | --- | --- |
|  |  | 1 | 2 | 3 | 4 | 5 |
|  | **Negative cognition** |  |  |  |  |  |
| Negative cognition | When things go wrong, I tend to give myself a hard time | 0.63 |  |  |  |  |
| Negative cognition | Sometimes I just can't let go of bad feelings | 0.59 |  |  |  |  |
| Negative cognition | I can't stop worrying about my problems | 0.57 |  |  |  |  |
| Negative cognition | If something upsets me it affects how I feel about everything | 0.54 |  |  |  |  |
| Negative cognition | I tend to think the worst is going to happen | 0.52 |  |  |  |  |
| Negative cognition | I worry about the future | 0.49 |  |  |  |  |
| Negative cognition | I dwell on the bad things that happen | 0.48 |  |  |  |  |
| Negative cognition | My feelings are out of my control | 0.47 |  |  |  |  |
| Social skills | I feel alone in the world | 0.47 |  |  |  |  |
| Negative cognition | I find it difficult to cope when things change unexpectedly | 0.44 |  |  |  |  |
| Negative cognition | When I make a mistake I feel that I am a hopeless person | 0.44 |  |  |  |  |
| Emotional insight | I like to think about why things happen the way they do | 0.43 |  |  |  |  |
| Empathy | I can understand how other people feel when they talk to me about their problems | 0.42 |  |  |  |  |
| Social skills | I feel that I am misunderstood | 0.42 |  |  |  |  |
| Negative cognition | I feel that I have little control over the things that happen to me | 0.38 |  |  |  |  |
| Emotional insight | I think about what things might be like for other people | 0.37 |  |  |  |  |
| Negative cognition | I worry about what people are thinking about me | 0.37 |  |  |  |  |
| Negative cognition | I get wound up about things | 0.34 |  |  |  |  |
| Negative cognition | I tend to get anxious in unfamiliar situations | 0.31 |  |  |  |  |
| Social skills | I enjoy spending time by myself |  |  |  |  |  |
|  | **Confidence (in self and future)** |  |  |  |  |  |
| Optimism | I feel hopeful about my life |  | 0.67 |  |  |  |
| Self-Confidence | I am confident that I can achieve what I set out to do |  | 0.65 |  |  |  |
| Self-Confidence | I feel confident that I can handle whatever comes my way |  | 0.64 |  |  |  |
| Self-Confidence | I feel good about myself |  | 0.56 |  |  |  |
| Optimism/ | My life has a sense of purpose |  | 0.54 |  |  |  |
| Optimism | I am a person who can go with the flow |  | 0.47 |  |  |  |
| Self-Confidence | I think about new activities or projects I would like to try |  | 0.45 |  |  |  |
| Self-Confidence | I feel confident to do things by myself |  | 0.43 |  |  |  |
| Self-Confidence | If I have a problem I can work it out |  | 0.43 |  |  |  |
| Self-Confidence | I feel stronger because of the problems I have faced |  | 0.39 |  |  |  |
| Emotional insight | If I get upset, I know how to make myself feel better |  | 0.38 |  |  | 0.30 |
| Optimism | I make plans for the future |  | 0.33 |  |  |  |
| Emotional insight | I have ways of getting rid of bad feelings |  | 0.31 |  |  |  |
| Social skills | I can stand up for myself when there is a problem |  |  |  |  |  |
| Emotional insight | I understand why I feel the way I do |  |  |  |  |  |
| Optimism | I use humour to help me feel better about problems |  |  |  |  |  |
| ARQ-Rev1 Scale | Factor | Factor | | | | |
|  |  | 1 | 2 | 3 | 4 | 5 |
| Optimism | Seeing the funny side of situations helps me when things get bad |  |  |  |  |  |
| Optimism | I can find positives even in bad situations |  |  |  |  |  |
| Social skills | I get a buzz out of meeting new people |  |  |  |  |  |
|  | **Empathy / Tolerance** |  |  |  |  |  |
| Empathy/Tolerance | I am patient with people who can't do things as well as I can |  |  | -0.55 |  |  |
| Empathy/Tolerance | I get frustrated when people make mistakes |  |  | 0.55 |  |  |
| Empathy/Tolerance | I am easily frustrated with people |  |  | 0.52 |  |  |
| Empathy/Tolerance | I expect people to live up to my standards |  |  | 0.49 |  |  |
| Negative cognition | I push myself too hard to do what everyone else does |  |  | 0.32 |  |  |
| Emotional insight | I am able to let go of things I can't control |  |  | -0.32 |  |  |
| Problem solving | I make quick decisions which I regret later |  |  |  |  |  |
| Emotional insight | I accept things that I can't change |  |  |  |  |  |
| Negative cognition | I am not happy unless things are perfect |  |  |  |  |  |
|  | **Social skills** |  |  |  |  |  |
| Social skills | I find it hard to express myself to others |  |  |  | 0.54 |  |
| Social skills | People come to me with their problems | 0.44 |  |  | -0.46 |  |
| Social skills | I feel helpless when faced with a problem |  |  |  | 0.41 |  |
| Social skills | I can share my personal thoughts with others |  |  |  | -0.39 |  |
| Problem solving | I find it hard to make important decisions | 0.32 |  |  | 0.37 |  |
| Negative cognition | I have trouble explaining how I am feeling | 0.30 |  |  | 0.36 |  |
| Social skills | I can express my opinions when I am in a group |  | 0.33 |  | -0.36 |  |
| Social skills | I feel pressured to do things because my friends do them |  |  | 0.31 | 0.33 |  |
| Problem solving | If something is becoming a problem I try to ignore it |  |  |  |  |  |
| Problem solving | If one approach to a problem doesn't work I find it hard |  |  |  |  |  |
|  | **Emotional insight** |  |  |  |  |  |
| Emotional insight | When I am feeling down, I take extra special care of myself |  |  |  |  | 0.48 |
| Emotional insight | I look for what I can learn out of bad things that happen |  |  |  |  | 0.44 |
| Emotional insight | I think things through carefully before making decisions |  |  |  |  | 0.42 |
| Emotional insight | I take it easy on myself when I am not feeling well |  |  |  |  | 0.41 |
| Problem solving | If I have a problem, I know there is someone I can talk to |  |  |  |  | 0.40 |
| Emotional insight | I slow down when things are going too fast |  |  |  |  | 0.40 |
| Problem solving | If I can't handle something I find help |  |  |  |  | 0.40 |
| Emotional insight | I can change my feelings by changing the way I see things |  |  |  |  | 0.37 |
| Emotional insight | I try to find meaning in the things that happen to me |  |  |  |  | 0.33 |
| Emotional insight | I can change the way I feel by changing the way I think |  |  |  |  |  |
| Social skills | I feel shy around people |  |  |  |  |  |

Note. Maximum Likelihood extraction, Oblimin rotation with Kaiser Normalisation,

Rotation converged in 20 iterations.
