# Supplementary material for: Development of a multi-dimensional measure of resilience in adolescents: the Adolescent Resilience Questionnaire
Source: BMC Med Res Methodol. 2011 Oct 5;11:134. doi: 10.1186/1471-2288-11-134 (PMC3204306; doi:10.1186/1471-2288-11-134)
Supplement: Additional file 7 — Study 2 Factor solution family domain. Study 2 output describing factor analysis of the family domain. Output includes the initial statistics for the two-factor solution with oblimin rotation, and the rotated factor loadings with the original conceptual scales, and factor developed scales described. [file 1471-2288-11-134-S7.DOCX]

**Additional file 7: Study 2. Factor output for the family domain**

Initial statistics for a two-factor solution of family domain with oblimin rotation (n = 451)

| Factor | Initial Eigenvalues | | | Rotation Sums of Squared Loadings(a) |
| --- | --- | --- | --- | --- |
|  | Total | % of Variance | Cumulative % | Total |
| 1 | 8.37 | 41.86 | 41.86 | 7.00 |
| 2 | 1.52 | 7.61 | 49.47 | 5.47 |
| 3 | 1.19 | 5.93 | 55.39 | 5.57 |
| 4 | 1.03 | 5.15 | 60.54 | 0.95 |
| 5 | 0.88 | 4.42 | 64.97 |  |
| 6 | 0.81 | 4.03 | 69.00 |  |
| 7 | 0.71 | 3.53 | 72.53 |  |
| 8 | 0.66 | 3.28 | 75.81 |  |
| 9 | 0.60 | 3.00 | 78.81 |  |
| 10 | 0.56 | 2.78 | 81.59 |  |
| 11 | 0.50 | 2.50 | 84.09 |  |
| 12 | 0.47 | 2.36 | 86.45 |  |
| 13 | 0.47 | 2.33 | 88.77 |  |
| 14 | 0.42 | 2.12 | 90.90 |  |
| 15 | 0.40 | 2.02 | 92.92 |  |
| 16 | 0.37 | 1.84 | 94.76 |  |
| 17 | 0.31 | 1.54 | 96.30 |  |
| 18 | 0.29 | 1.46 | 97.75 |  |
| 19 | 0.25 | 1.23 | 98.99 |  |
| 20 | 0.20 | 1.01 | 100.00 |  |

Extraction Method: Maximum Likelihood.

a. When factors are correlated, sums of squared loadings cannot be added to obtain a total variance.

Factor solution for the family domain (n = 451)

| ARQ-Rev1 Scale^a^ | Factor^b^ | 1 | 2 |
| --- | --- | --- | --- |
|  | **Connectedness** |  |  |
| Connectedness | I do fun things with my family | 0.88 |  |
| Connectedness | We do things together as a family | 0.81 |  |
| Connectedness | My family understands my needs | 0.71 |  |
| Connectedness | I enjoy spending time with my family | 0.69 |  |
| Connectedness | I get to spend enough time with my family | 0.68 |  |
| Connectedness | My family is a safe place when things seem to be falling apart | 0.67 |  |
| Availability | I have a say in family decisions | 0.66 |  |
| Connectedness | My family helps me to believe in myself and my abilities | 0.65 |  |
| Connectedness | My family listens to me | 0.64 |  |
| Connectedness | My family provides me with emotional support | 0.58 |  |
| Connectedness | My parents trust me to look after myself | 0.47 |  |
| Connectedness | People in my family expect too much of me | -0.45 |  |
| Connectedness | I can be honest with my family about how I feel | 0.42 | -0.35 |
| Availability | People in my family are too busy to pay attention to me | -0.40 |  |
| Connectedness | I don't feel loved by my family | -0.37 |  |
| Connectedness | My family talks about problems we are having | 0.34 |  |
|  | **Availability** |  |  |
| Availability | There is someone in my family I can talk to about anything |  | -0.92 |
| Availability | If I have a problem there is someone in my family I can talk to |  | -0.79 |
| Availability | There is someone in my family that I feel particularly close to |  | -0.50 |
| Connectedness | My mum or dad is over protective of me |  |  |

a. Column one identifies the conceptual scale each item was associated with.

b. Maximum Likelihood extraction and Oblimin rotation with Kaiser normalisation.
