# Supplementary material for: Development of a multi-dimensional measure of resilience in adolescents: the Adolescent Resilience Questionnaire
Source: BMC Med Res Methodol. 2011 Oct 5;11:134. doi: 10.1186/1471-2288-11-134 (PMC3204306; doi:10.1186/1471-2288-11-134)
Supplement: Additional file 8 — Study 2 Factor solution peer domain. Study 2 output describing factor analysis of the peer domain. Output includes the initial statistics for the two-factor solution with oblimin rotation, and the rotated factor loadings with the original conceptual scales, and factor developed scales described. [file 1471-2288-11-134-S8.DOCX]

**Additional file 8: Study 2. Factor output for the peer domain**

Initial statistics for a two-factor solution with oblimin rotation (n = 451)

Total variance explained

| Factor | Initial Eigenvalues | | | Rotation Sums of Squared Loadings (a) |
| --- | --- | --- | --- | --- |
|  | Total | % of Variance | Cumulative % | Total |
| 1 | 3.95 | 35.90 | 35.90 | 3.09 |
| 2 | 1.32 | 11.99 | 47.89 | 2.32 |
| 3 | 0.94 | 8.59 | 56.48 |  |
| 4 | 0.89 | 8.06 | 64.54 |  |
| 5 | 0.75 | 6.80 | 71.34 |  |
| 6 | 0.67 | 6.10 | 77.44 |  |
| 7 | 0.61 | 5.52 | 82.96 |  |
| 8 | 0.57 | 5.19 | 88.15 |  |
| 9 | 0.51 | 4.64 | 92.79 |  |
| 10 | 0.42 | 3.86 | 96.65 |  |
| 11 | 0.37 | 3.35 | 100.00 |  |

Extraction Method: Maximum Likelihood.

a. When factors are correlated, sums of squared loadings cannot be added to obtain a total variance.

Factor solution for the peer domain (n = 451)

| ARQ-Rev1 Scale^a^ | Factor^b^ | Factor | |
| --- | --- | --- | --- |
|  |  | 1 | 2 |
|  | **Connectedness** |  |  |
| Connectedness | When I am down I have friends that help cheer me up | 0.78 |  |
| Availability | I have a group of friends that I keep in touch with regularly | 0.65 |  |
| Availability | I have a friend I can trust with my private thoughts and feelings | 0.65 |  |
| Connectedness | I have friends who make me laugh | 0.59 |  |
| Connectedness | I enjoy being around people my age | 0.57 |  |
| Availability | I get to spend enough time with my friends | 0.41 |  |
| Connectedness | I feel confident around people my age | 0.40 |  |
|  | **Availability (negative)** |  |  |
| Connectedness | I feel left out of things |  | 0.82 |
| Availability | I wish I had more friends I felt close to |  | 0.55 |
| Availability | I find it hard making friends |  | 0.46 |
| Connectedness | My friends get me into trouble |  |  |

a. Column one identifies the conceptual scale each item was associated with.

b. Maximum Likelihood extraction and Oblimin rotation with Kaiser normalisation.
