# Supplementary material for: Development of a multi-dimensional measure of resilience in adolescents: the Adolescent Resilience Questionnaire
Source: BMC Med Res Methodol. 2011 Oct 5;11:134. doi: 10.1186/1471-2288-11-134 (PMC3204306; doi:10.1186/1471-2288-11-134)
Supplement: Additional file 9 — Study 2 Factor solution school domain. Study 2 output describing factor analysis of the school domain. Output includes the initial statistics for the two-factor solution with oblimin rotation, and the rotated factor loadings with the original conceptual scales, and factor developed scales described. [file 1471-2288-11-134-S9.DOCX]

**Additional file 9: Study 2. Factor output for the school domain**

Initial statistics for a two-factor solution with oblimin rotation (n = 451)

| Total Variance Explained | | | | |
| --- | --- | --- | --- | --- |
| Factor | Initial Eigenvalues | | | Rotation Sums of Squared Loadings^a^ |
|  | Total | % of Variance | Cumulative % | Total |
| 1 | 4.59 | 30.62 | 30.62 | 3.65 |
| 2 | 1.46 | 9.74 | 40.36 | 1.45 |
| 3 | 1.35 | 8.98 | 49.34 | 2.67 |
| 4 | 1.01 | 6.70 | 56.04 |  |
| 5 | 0.93 | 6.19 | 62.23 |  |
| 6 | 0.86 | 5.72 | 67.94 |  |
| 7 | 0.78 | 5.21 | 73.15 |  |
| 8 | 0.68 | 4.54 | 77.69 |  |
| 9 | 0.67 | 4.44 | 82.13 |  |
| 10 | 0.57 | 3.78 | 85.90 |  |
| 11 | 0.55 | 3.69 | 89.60 |  |
| 12 | 0.47 | 3.16 | 92.75 |  |
| 13 | 0.43 | 2.86 | 95.61 |  |
| 14 | 0.38 | 2.50 | 98.11 |  |
| 15 | 0.28 | 1.89 | 100.00 |  |

Extraction Method: Maximum Likelihood.

a. When factors are correlated, sums of squared loadings cannot be added to obtain a total variance.

Factor solution for the school domain (n = 451)

| ARQ-Rev1 scale^a^ | ARQ-Rev1 Factor^b^ | Factor | |
| --- | --- | --- | --- |
|  |  | 1 | 2 |
|  | **Supportive environment** |  |  |
| Supportive environment | My teachers are caring and supportive of me | 0.83 |  |
| Supportive environment | Teachers in my school are caring | 0.74 |  |
| Supportive environment | My teachers provide me with extra help if I need it | 0.66 |  |
| Supportive environment | I have a teacher that I feel looks out for me | 0.63 |  |
| Supportive environment | My teachers notice when I am doing a good job and let me know about it | 0.58 |  |
| Supportive environment | There is an adult at school who I could talk to if I had a personal problem | 0.49 |  |
| Supportive environment | I feel that what I say counts at school | 0.48 |  |
| Supportive environment | At school students help to decide and plan things like school activities and events | 0.31 |  |
| Connectedness | I get involved with school activities | 0.31 |  |
| Connectedness | I feel included by other students at school |  |  |
|  | **Connectedness** |  |  |
| Connectedness | I hate going to school |  | 0.77 |
| Connectedness | I am bored at school |  | 0.66 |
| Connectedness | I try hard in school |  | -0.40 |
| Supportive environment | My teachers expect too much of me |  | 0.33 |
| Connectedness | I get teased at school |  |  |

a. Column one identifies the conceptual scale each item was associated with.

b. Maximum Likelihood extraction and Oblimin rotation with Kaiser normalisation.
