# Supplementary material for: Development of a multi-dimensional measure of resilience in adolescents: the Adolescent Resilience Questionnaire
Source: BMC Med Res Methodol. 2011 Oct 5;11:134. doi: 10.1186/1471-2288-11-134 (PMC3204306; doi:10.1186/1471-2288-11-134)
Supplement: Additional file 10 — Study 2 Factor solution community domain. Study 2 output describing factor analysis of the community domain. Output includes the initial statistics for the two-factor solution with oblimin rotation, and the rotated factor loadings with the original conceptual scales, and factor developed scales described. [file 1471-2288-11-134-S10.DOCX]

**Additional file 10: Study 2. Factor output for the community domain**

Initial statistics for a two-factor solution with oblimin rotation (n = 451)

| Factor | Initial Eigenvalues | | | Rotation Sums of Squared |
| --- | --- | --- | --- | --- |
|  | Total | % of Variance | Cumulative % | Loadings(a) Total |
| 1 | 6.48 | 43.25 | 43.25 | 6.01 |
| 2 | 1.30 | 8.73 | 51.98 | 1.42 |
| 3 | 1.06 | 7.13 | 59.11 |  |
| 4 | 0.90 | 6.01 | 65.12 |  |
| 5 | 0.83 | 5.58 | 70.70 |  |
| 6 | 0.76 | 5.08 | 75.79 |  |
| 7 | 0.72 | 4.82 | 80.61 |  |
| 8 | 0.64 | 4.27 | 84.88 |  |
| 9 | 0.45 | 3.07 | 87.94 |  |
| 10 | 0.44 | 2.97 | 90.91 |  |
| 11 | 0.35 | 2.34 | 93.25 |  |
| 12 | 0.28 | 1.92 | 95.17 |  |
| 13 | 0.27 | 1.80 | 96.97 |  |
| 14 | 0.24 | 1.66 | 98.63 |  |
| 15 | 0.20 | 1.37 | 100.00 |  |

Extraction Method: Maximum Likelihood.

a. When factors are correlated, sums of squared loadings cannot be added to obtain a total variance.

Factor solution for the community domain (n = 451)

|  | Factor^a^ | |
| --- | --- | --- |
|  | 1 | 2 |
| I trust the people in my neighbourhood | 0.90 |  |
| People in my neighbourhood are caring | 0.83 |  |
| The people in my neighbourhood treat other people fairly | 0.80 |  |
| I like my neighbourhood | 0.80 |  |
| The people in my neighbourhood look out for me | 0.77 |  |
| I like the people in my neighbourhood | 0.75 |  |
| The people in my neighbourhood look out for one another | 0.73 |  |
| People in my neighbourhood go out of their way to help | 0.70 |  |
| People in my neighbourhood know me personally | 0.51 | 0.36 |
| There is an adult in my neighbourhood I could talk to if I had | 0.42 |  |
| Young people have a say in what happens in our neighbourhood | 0.40 |  |
| I get involved in social groups in my neighbourhood | 0.31 |  |
| I feel isolated in my neighbourhood |  |  |
| If I did something wrong people in my neighbourhood would find out |  | 0.36 |
| People in my neighbourhood keep to themselves |  | -0.30 |

a. Maximum Likelihood extraction and Oblimin rotation with Kaiser normalisation.
